# Effect of In Situ Synthesized Al_2_O_3_ and TiC on the Microstructure and Properties of 6061 Aluminum Matrix Composites

**DOI:** 10.3390/ma19020308

**Published:** 2026-01-12

**Authors:** Wei Long, Jiaxin Zhou, Xinbin Hu, Sheng Liu, Wenming Jiang

**Affiliations:** 1 School of Materials and Chemical Engineering, Hubei University of Technology, Wuhan 430068, China; maillong1982@126.com (W.L.); 15549876985@163.com (J.Z.); huxbshu@163.com (X.H.); 2 Key Laboratory of Green Materials for Light Industry of Hubei Provincial, Wuhan 430068, China; 3 Hubei Engineering Laboratory of Automotive Lightweight Materials and Processing, Wuhan 430068, China; 4 State Key Laboratory of Materials Processing and Die & Mould Technology, School of Materials Science and Engineering, Huazhong University of Science and Technology, Wuhan 430074, China

**Keywords:** in situ synthesis, composites, sintering temperature, ceramic particles, wear property

## Abstract

Al_2_O_3_-TiC/6061Al composites were fabricated via in situ powder metallurgy using 6061 Al, TiO_2_, and graphite powders as starting materials. The effects of sintering temperature and ceramic particle content on the microstructure and mechanical properties of the composites were investigated. The wear performance of composites sintered at 1200 °C with varying ceramic particle content was also examined. The results indicate that the microstructure of the composite varied with the sintering temperature. At 1000 °C and 1100 °C, the microstructure primarily consisted of Al_3_Ti, Al_2_O_3_, and TiC phases. At 1200 °C and 1250 °C, the microstructure was predominantly composed of Al_2_O_3_ and TiC phases. The 6061 Al-12% (TiO_2_ + C) composite sintered at 1200 °C exhibited a tensile strength of 246 MPa, an elongation of 12.7%, and a microhardness of 104.2 HV_0.1_. Regarding wear performance, the wear behavior of the composites under different loads at 1200 °C was studied. Under a 30 N load, the 6061 Al-12% (TiO_2_ + C) composite demonstrated the lowest friction coefficient and wear rate, measured at 0.253 and 0.396 mm^3^·N^−1^·m^−1^, respectively. Analysis of the worn surface morphology under a 30 N load indicates that the dominant wear mechanism for the 6061 aluminum alloy is delamination wear, whereas for the 6061 Al-12% (TiO_2_ + C) composite, it is primarily abrasive wear.

## 1. Introduction

Aluminum Matrix Composites (AMCs), as a critical component of advanced lightweight structural material systems, have demonstrated broad application prospects in key lightweight fields such as aerospace, national defense, and transportation due to their excellent specific strength, high specific stiffness, outstanding wear resistance, and good elevated-temperature properties [1,2,3]. Among them, 6061 aluminum alloy is widely selected as the matrix material for AMCs owing to its good comprehensive mechanical properties, excellent processability, and high commercial availability [4,5]. However, under extreme service conditions such as high temperature, high load, or severe wear, its inherent absolute strength, hardness, and high-temperature stability still struggle to meet the requirements of high-performance components, showing significant shortcomings particularly in wear-resistant parts. Consequently, introducing high-hardness, high-modulus ceramic particles to fabricate composites has become an important pathway to overcome these performance limitations [6,7].

Among various reinforcing particles, alumina (Al_2_O_3_) and titanium carbide (TiC) are recognized as ideal candidate reinforcements due to their high melting points, high hardness, exceptional wear resistance, and excellent thermal stability [8,9]. Currently, preparation processes for AMCs can be broadly divided into two main categories: ex situ (externally added) methods [10,11,12,13] and in situ (self-formed) methods [14,15,16,17,18]. Although conventional ex situ methods are relatively straightforward, they often encounter issues such as interfacial contamination, weak interfacial bonding between the reinforcement and the metal matrix, and agglomeration of the reinforcing particles, making it difficult to achieve uniform dispersion at the sub-micron level, thus limiting the full exploitation of the material properties. For instance, in AA6061/TiC composites prepared by Moses et al. [19] via stir casting, the average TiC particle size was about 2 μm but showed significant segregation; Shaikh et al. [20] reported composites fabricated by powder metallurgy where the reinforcement size reached up to 40 μm, severely compromising the strengthening effect. These cases illustrate the inherent limitations of the direct addition of ceramic particles in terms of interfacial bond quality and distribution homogeneity.

In contrast, in situ synthesis technology generates reinforcing particles directly through chemical reactions between components within the matrix, enabling the formation of reinforcements with clean surfaces, strong interfacial bonding with the matrix, and fine-scale (typically nano- or submicron-sized) uniform distribution. This approach significantly enhances the overall performance of composites by producing thermodynamically stable, finely dispersed reinforcements that avoid the common issues of particle agglomeration and weak interfacial bonds found in ex situ methods. Among various reaction systems, the Al-TiO_2_-C system [21,22,23,24,25] is particularly attractive, as it allows the simultaneous generation of Al_2_O_3_ and TiC ceramic particles directly within the aluminum matrix in a single sintering step. Researchers have conducted preliminary explorations in this area: for instance, Zhu et al. [26] investigated the reaction pathway, activation energy, and mechanical properties of the Al-TiO_2_-C system, reporting a tensile strength of 351.8 MPa and an elongation of 5.6% at a C/TiO_2_ molar ratio of 1. Xia et al. [27] studied the effect of TiC-Al_2_O_3_ ceramic reinforcements on ZL101 composites, demonstrating that the microhardness of in situ synthesized Al_2_O_3_-TiC/ZL101 composites increased significantly from 93 HV (base alloy) to 162 HV. Odhiambo et al. [28] synthesized Al_2_O_3_, TiB_2_, and TiC submicron ceramic particle-reinforced aluminum composites via reaction sintering of Al, TiO_2_, and B_4_C powder mixtures. Their results showed that with 25% ceramic content, the composites achieved an average tensile strength of 425 MPa and a yield strength of 302 MPa. These outcomes are markedly superior to those achieved by conventional ex situ methods, where reinforcements often exhibit sizes in the tens of micrometers and significant aggregation.

However, in current research, there are few reports that systematically discuss the in situ synthesis of Al_2_O_3_ and TiC particles using 6061 aluminum alloy as the matrix via powder metallurgy. Furthermore, most literature primarily focuses on the reaction mechanisms and mechanical properties of the materials, often lacking a systematic analysis of other key properties, such as wear resistance, which limits the application of these materials in other fields. Therefore, this study selects 6061 aluminum alloy powder, TiO_2_ powder, and graphite powder as raw materials, combining powder metallurgy with in situ synthesis technology to fabricate a composite material in which Al_2_O_3_ and TiC particles are synthesized in situ within the 6061 aluminum matrix through the Al-TiO_2_-C reaction system. This work emphasizes investigating the influence of sintering temperature on the microstructure, mechanical properties, and wear resistance of the in situ synthesized composites. It is expected to achieve effective incorporation of Al_2_O_3_ and TiC particles within the 6061 aluminum matrix, providing experimental basis and theoretical support for the design and preparation of high-performance aluminum matrix composites.

## 2. Experimental Procedures

### 2.1. Materials

The matrix material consisted of 6061 Al alloy powder (purity > 99%, Sinopsin Group Chemical Reagents Co., Ltd., Nanjing, China, particle size: 500 mesh). The reinforcing precursors were TiO_2_ powder (purity ≥ 99.8%, supplied by Shanghai Yaoge Alloy Material Co., Ltd., Shanghai, China, rutile phase, average particle size: 1 μm) and graphite powder (purity ≥ 99%, provided by Guangzhou Metal Metallurgy Co., Ltd., Guangzhou, China, average particle size: 5 μm). The primary chemical composition of the 6061 Al alloy powder is listed in Table 1.

### 2.2. Preparation of Materials

The initial powder ratios for the experiments were calculated based on the overall reaction Equation (1), where x represents the excess Al. The specific compositions of different samples are listed in Table 2. According to Equation (1), composites with (TiO_2_ + graphite) contents of 0, 6, 9, and 12 wt% were prepared.

(x + 4)Al + 3TiO_2_ + 3C = xAl + 2Al_2_O_3_ + 3TiC (1)

**Figure 1:**
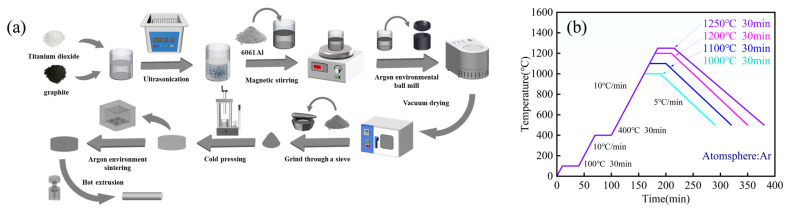
Experimental schematic diagram of Al_2_O_3_-TiC/6061 aluminum matrix composites: (**a**) Experimental flowchart; (**b**) Sintering process curve.

The fabrication process of the composites is illustrated in Figure 1a. Initially, TiO_2_ and graphite powders were pretreated using ultrasonic dispersion to mitigate agglomeration. The 6061 Al powder was then added to the mixture, followed by preliminary dispersion via magnetic stirring. The mixed powders were subsequently subjected to wet ball milling in a planetary ball mill. Anhydrous ethanol was used as the milling medium, with 1 wt% stearic acid added as a process control agent. The ball milling was conducted under an argon atmosphere at a rotation speed of 220 r/min, adopting an intermittent operation mode (15 min of milling followed by 5 min of pause) to prevent overheating and suppress particle cold welding. The total milling time was 6 h.

The ball-milled products were vacuum-dried at 60 °C, ground in an agate mortar, and sieved. Cylindrical green compacts were fabricated via cold isostatic pressing at a pressure of 200 MPa. Subsequently, a step-sintering process was conducted in an argon atmosphere: the compacts were first held at 100 °C for 30 min for dehydration, followed by a 30 min holding at 400 °C to remove residual stearic acid, and finally sintered at temperatures ranging from 1000 to 1250 °C for 30 min (The sintering profile is illustrated in Figure 1b). The selection of a sintering temperature in the range of 1000–1250 °C is primarily to initiate and complete the self-propagating high-temperature synthesis (SHS) reaction (1).

To enhance the densification of the materials, the sintered specimens were subjected to hot extrusion at 480 °C with an extrusion ratio of 10:1 and a extrusion speed of 1 mm/s. For comparison, a control group of 6061 Al alloy was prepared under identical conditions. The volume fractions of the reinforcing phases in the samples sintered at 1200 °C and 1250 °C followed by hot extrusion are listed in Table 3. The results indicate that the volume fractions of Al_2_O_3_ and TiC in the samples gradually increased with the rising content of TiO_2_ and graphite.

The phase composition of the samples was identified by X-ray diffraction (XRD, Bruker D8 Advance, Bruker, Billerica, MA, USA) using Cu-Kα radiation at a scanning rate of 5°/min over the 2θ range of 10° to 90°. The microstructure of the composites was characterized by scanning electron microscopy (SEM, ZEISS Sigma 300, ZEISS, Oberkochen, Germany) coupled with energy-dispersive X-ray spectroscopy (EDS). Tensile tests were conducted on the hot-extruded samples at room temperature using a universal testing machine (Shimadzu UH-300kNI, Shimadzu, Kyoto, Japan) with a crosshead speed of 1 mm/min, corresponding to an initial strain rate of 4.90 × 10^−4^ s^−4^. Three specimens were tested for each condition, and the average value is reported. Vickers hardness was measured using a microhardness tester (UML VMHT, Walter Uhl technische Mikroskopie GmbH & Co. KG, Aßlar, Germany) with a load of 0.3 kg applied for a dwell time of 15 s; the final value for each sample represents the average of 10 indents. Dry sliding wear tests were performed on a high-frequency reciprocating friction and wear tester (MFT-EC4000, Lanzhou Huahui Instrument Technology Co., Ltd., Lanzhou, China). The 3D morphology of the wear tracks was examined using an optical profiler (Bruker Contour GT-K 3D, Bruker, Ettlingen, Germany).

## 3. Results and Discussion

### 3.1. Morphology Analysis of Ball-Milled Powders

**Figure 2:**
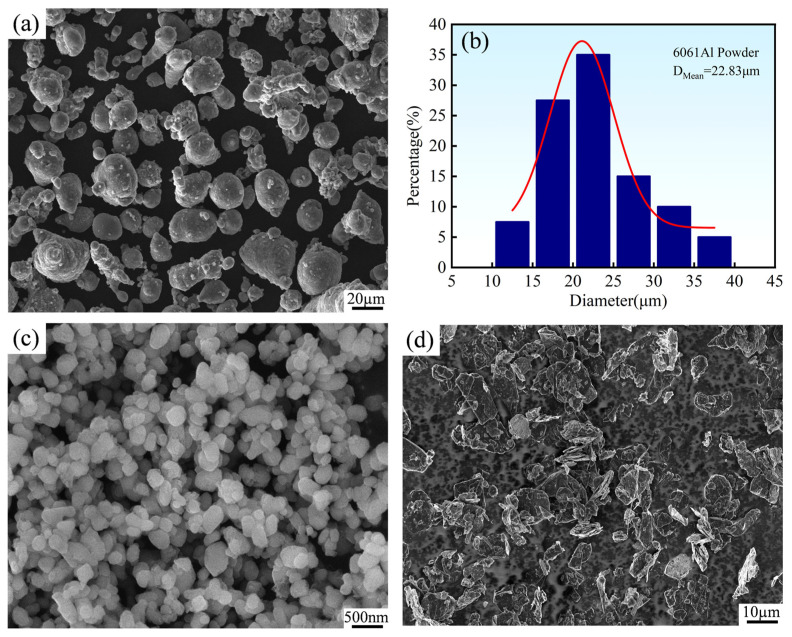
Microstructure and particle size distribution of the raw powders: (**a**) 6061 Al; (**b**) particle size distribution of 6061 Al; (**c**) TiO_2_; (**d**) graphite.

Figure 2 presents the microscopic morphology of the raw powders and the particle size distribution characteristics of the 6061 aluminum alloy powder. It can be observed from Figure 2a that the 6061 Al powder exhibits an irregular spherical morphology. Figure 2b further shows the particle size distribution curve of the 6061 Al powder, which approximates a normal distribution with its peak concentrated in the range of 20–25 μm and an average particle size of 22.83 μm, indicating relatively uniform powder particle sizes. Additionally, Figure 2c reveals the spherical particle morphology of the TiO_2_ powder, but with noticeable agglomeration. Figure 2d displays the irregular flake-like structure of the graphite powder.

**Figure 3:**
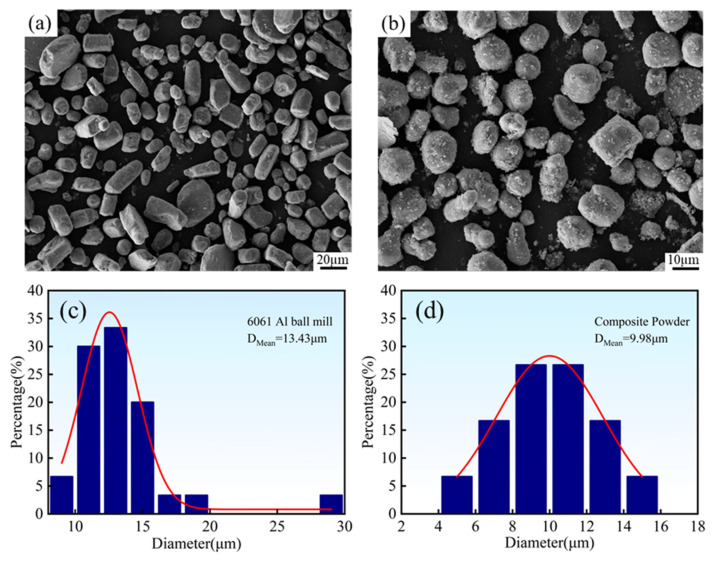
Morphology and particle size distribution of the ball-milled powders: (**a**) 6061 Al powder; (**b**) mixed powder; (**c**) particle size distribution of 6061 Al powder; (**d**) particle size distribution of the mixed powder.

Figure 3 illustrates the microscopic morphology and particle size distribution results of the powders after ball milling treatment. It is evident that the ball milling process significantly improves the dispersibility of the powders, resulting in a uniform microstructure. Particle size analysis indicates that the average particle size of the ball-milled 6061 Al powder decreases to 13.43 μm, while the average particle size of the composite powder is further reduced to 9.98 μm. Compared with the unmilled 6061 Al powder (22.83 μm), the ball-milled powders demonstrate a significant reduction in particle size, which fully confirms the effectiveness of the ball milling and mixing process.

### 3.2. XRD Analysis

**Figure 4:**
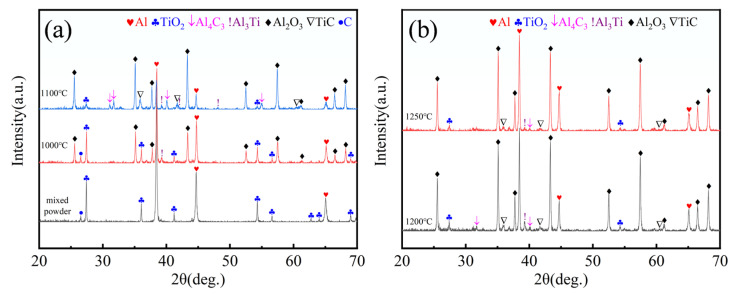
XRD patterns of ball-milled powder and sintered powder at different temperatures: (**a**) Ball-milled powder, sintered powder at 1000 °C and 1100 °C; (**b**) Sintered powders at 1200 °C and 1250 °C.

Figure 4 shows the XRD patterns of the mixed powders after ball milling and those sintered at different temperatures. It can be observed that the ball-milled powder primarily exhibits diffraction peaks corresponding to Al and TiO_2_. No diffraction peak for carbon is detected due to its low content being below the instrument’s detection limit, and no impurity peaks are present in the patterns, indicating that the ball-milling process did not introduce significant contamination and proving the rationality and feasibility of the employed ball-milling procedure.

Based on thermodynamic and kinetic analyses, reaction Equation (1) can be decomposed into multiple steps Equations (2)–(4). The stepwise nature of this reaction pathway has been reported in the literature [27,28]. Combined with the XRD patterns, it is evident that at 1000 °C, the diffraction peaks of TiO_2_ weaken, while peaks corresponding to Al_2_O_3_ and Al_3_Ti emerge, indicating the occurrence of the redox reaction represented by Equation (2). When the temperature increases to 1100 °C, the TiO_2_ peaks further weaken, the Al_2_O_3_ and Al_3_Ti peaks intensify, and diffraction peaks of Al_4_C_3_ and TiC begin to appear. This suggests that the reaction described by Equation (2) continues to proceed, while the carbothermal reduction reaction Equation (3) and the reaction in Equation (4) occur simultaneously. As the temperature reaches 1200 °C and 1250 °C, the TiO_2_ is completely consumed, and the TiC diffraction peaks become significantly stronger, indicating the reaction approaches thermodynamic equilibrium with TiC particles growing gradually. Ultimately, a 6061 Al matrix composite reinforced with Al_2_O_3_ and TiC ceramic particles is formed.

13Al + 3TiO_2_ = 2Al_2_O_3_ + 3Al_3_Ti (2)

4Al + 3C = Al_4_C_3_ (3)

3Al_3_Ti+ Al_4_C_3_ = 3TiC + 13Al (4)

### 3.3. Density and Microstructure of Composite Materials

The parameters related to density and porosity of the samples sintered at 1200 °C and 1250 °C followed by hot extrusion are presented in Table 4. As can be seen from the table, at different sintering temperatures, the density of the materials gradually decreases and the porosity gradually increases with higher ceramic particle content. This may be attributed to pores existing in the green compact as well as those formed during the chemical reaction that produces the final ceramic particles [28]. Compared to the specimens sintered at 1200 °C, those with the same composition sintered at 1250 °C exhibit higher porosity. This indicates that increasing the sintering temperature does not improve densification but rather leads to greater porosity [29].

**Figure 5:**
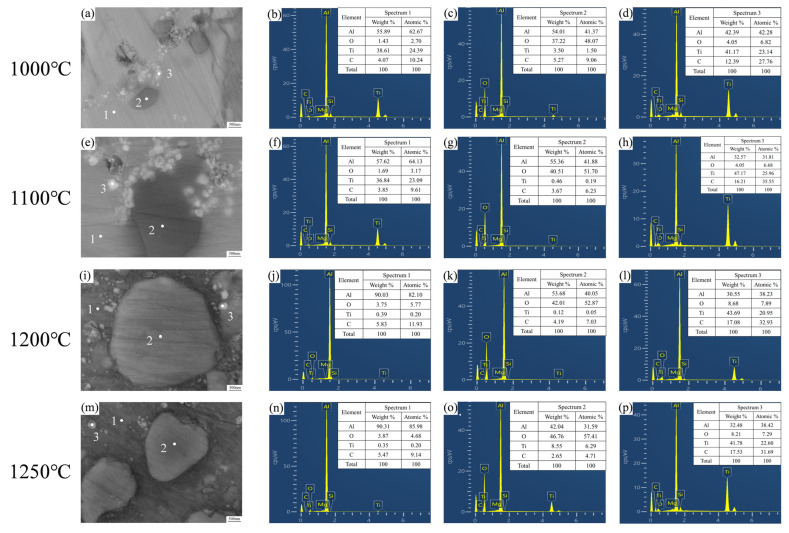
Microstructure and corresponding EDS analysis of the A12 samples sintered at different temperatures followed by hot extrusion: (**a**–**d**) 1000 °C; (**e**–**h**) 1100 °C (**i**–**l**) 1200 °C; (**m**–**p**) 1250 °C. (Due to the relatively low measured contents of Mg and Si elements in EDS, the Mg and Si elements have been omitted from the composition tables in Figure 5. The remaining elements have been normalized.).

Figure 5 shows the microstructural morphology and the corresponding EDS results of the A12 samples after sintering at various temperatures and subsequent hot extrusion treatment. Scanning electron microscopy (SEM) images at 1000 °C and 1100 °C (Figure 5a,e) reveal that white particles exhibit slight agglomeration in local regions. At 1000 °C, the EDS analysis for point 1 shows mass fractions of 62.67% for Al and 24.39% for Ti, indicating a composition close to the theoretical value of Al_3_Ti. The composition at point 2 is 41.37% Al and 48.07% O, consistent with Al_2_O_3_ stoichiometry. Point 3 exhibits mass fractions of 23.14% Ti and 27.76% C, which basically matches the composition of TiC. The specific EDS results are shown in Figure 5b–d, suggesting the presence of Al_3_Ti, Al_2_O_3_, and TiC particles in the microstructure sintered at 1000 °C. At 1100 °C, the compositions of the detected points correspond to Al_3_Ti, Al_2_O_3_, and TiC particles, respectively, similar to those at 1000 °C, with specific EDS results shown in Figure 5f–h. The phase identification results at both temperatures are consistent with the trend observed in the XRD analysis. However, TiC was not detected by XRD at 1000 °C, while fine TiC particles were observed under SEM. This is attributed to the initial stage of reaction (4), resulting in TiC particles with small size and low content. As the temperature increases to 1100 °C, the TiC particle size increases significantly, and the reaction proceeds further, leading to clear identification of TiC peaks in the XRD pattern. These microstructural characteristics agree with the findings reported by Duange et al. [30].

At 1200 °C and 1250 °C, SEM images (Figure 5i,m) indicate that the particles are distributed in the matrix without significant agglomeration. The results at 1200 °C show that the mass fraction of aluminum at point 1 is 82.10%, indicating that this region is the aluminum matrix. Points 2 and 3 correspond to Al_2_O_3_ and TiC particles, respectively, with specific EDS data provided in Figure 5j–l. This analysis confirms that only Al_2_O_3_ and TiC particles are present in the microstructure sintered at 1200 °C. At 1250 °C, the compositional distribution of the detected points is consistent with that at 1200 °C, corresponding to the aluminum matrix, Al_2_O_3_, and TiC, respectively. The corresponding EDS results are shown in Figure 5n–p. The identification results are generally consistent with the trend observed in the X-ray diffraction (XRD) analysis. However, very weak peaks of Al_3_Ti and Al_4_C_3_ are still detectable in the XRD patterns, which might be attributed to incomplete reactions due to differences in powder flowability during the sintering process, leading to the retention of a small amount of Al_3_Ti and Al_4_C_3_.

Comparative analysis of the microstructures of the samples sintered at different temperatures revealed that the average particle size of Al_2_O_3_ (approximately 2–4 μm) was significantly larger than that of TiC particles (around 200 nm). This phenomenon primarily stems from the notable differences in the nucleation kinetics of the two phases during the sintering process. Specifically, Al_2_O_3_ particles undergo homogeneous nucleation at a relatively low temperature of 800 °C (or even lower), while TiC nucleation requires a higher temperature, exceeding 900 °C. This result is consistent with the findings of Odhiambo et al. [28], who observed the formation of Al_2_O_3_ and TiC at approximately 700 °C and 900 °C, respectively.

### 3.4. Mechanical Properties

**Figure 6:**
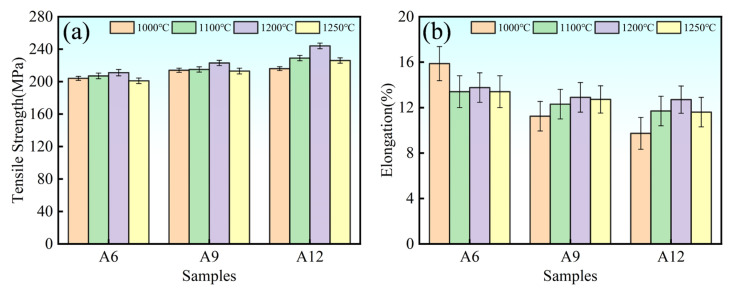
Samples sintered at different temperatures and after subsequent hot extrusion: (**a**) Tensile strength; (**b**) Elongation.

Figure 6 shows the variations in the ultimate tensile strength (UTS) and elongation of samples with different compositions sintered at various temperatures and subsequently subjected to hot extrusion treatment. The results indicate that the UTS of the composites increases monotonically with the ceramic particle content at a given sintering temperature. Meanwhile, the UTS first increases and then decreases as the sintering temperature rises. The A12 sample sintered at 1200 °C exhibited the optimal tensile properties, with a tensile strength of 246 MPa and an elongation of 12.7%. In comparison, the A0 reference sample showed a tensile strength of 188 MPa and an elongation of 26.8%. The tensile strength of the A12 sample is 30.8% higher than that of the A0 sample, while still retaining a reasonably good elongation. This suggests that at this sintering temperature, the strengthening effect of the ceramic particles is most pronounced, effectively enhancing the strength without severely compromising the ductility.

The underlying reasons for this trend can be elucidated by combining the analysis of Figure 5. At 1000 °C, the composite microstructure primarily consists of Al_2_O_3_, Al_3_Ti, and TiC. The strength is predominantly governed by thermal mismatch strengthening and Orowan strengthening mechanisms [31,32]. The significant difference in the coefficients of thermal expansion (CTE) between the Al_2_O_3_ (CTE = 7.3 × 10^−6^ K^−1^), Al_3_Ti (CTE = 19 × 10^−6^ K^−1^), and TiC (CTE = 7.4 × 10^−6^ K^−1^) particles and the 6061 Al matrix (CTE = 23.6 × 10^−6^ K^−1^) generates residual stress fields upon cooling, which hinder dislocation glide, resulting in thermal mismatch strengthening. Furthermore, the sub-micron Al_2_O_3_ particles strengthen the material via the Orowan mechanism by pinning grain boundaries and obstructing dislocation motion. However, the weak interfacial bonding between the Al_3_Ti phase and the matrix tends to form micro-crack initiation sites, thereby weakening the synergistic strengthening effect. Concurrently, the primary TiC particles are unstable, non-uniformly distributed, and exhibit agglomeration, which can degrade the material’s properties. At 1100 °C, the strength improvement is limited, primarily due to microstructural inhomogeneity. The Al_2_O_3_ and Al_3_Ti particles coarsen, and the diffusion of TiC particles remains insufficient, leading to weakened interfacial bonding with the matrix and consequently limited performance enhancement.

At 1200 °C, the final microstructure comprises TiC and Al_2_O_3_ particles. The significant CTE mismatch between these ceramic particles (TiC, Al_2_O_3_) and the 6061 Al matrix again contributes to thermal mismatch strengthening. Moreover, the nano-scale TiC particles enhance the material’s strength through the Orowan mechanism by effectively pinning dislocations and restricting grain boundary sliding. The synergistic interaction of these multiple strengthening mechanisms collectively and significantly enhances the overall mechanical properties, resulting in the optimal performance observed. When the sintering temperature reaches 1250 °C, the elevated temperature may promote rapid coarsening of the aluminum matrix and ceramic particles, leading to a decline in mechanical properties [33,34]. Furthermore, excessively high temperatures could cause partial melting or a significant reduction in the viscosity of the aluminum matrix. This would alter the shape of the preform, resulting in uneven distribution of the liquid phase or excessive volatilization, thereby introducing pores and defects that reduce the strength and densification of the material [35,36].

### 3.5. Hardness and Tribological Properties

Hardness is defined as the ability of a material to resist indentation or scratching. In aluminum alloys, hardness is closely related to their microstructure, such as strengthening particles. Wear resistance refers to the ability of a material to resist material loss caused by friction. Hardness is a key factor influencing wear resistance: under similar conditions, aluminum alloys with high hardness can more effectively resist the penetration and ploughing of abrasive particles, thereby reducing wear and improving wear resistance.

**Figure 7:**
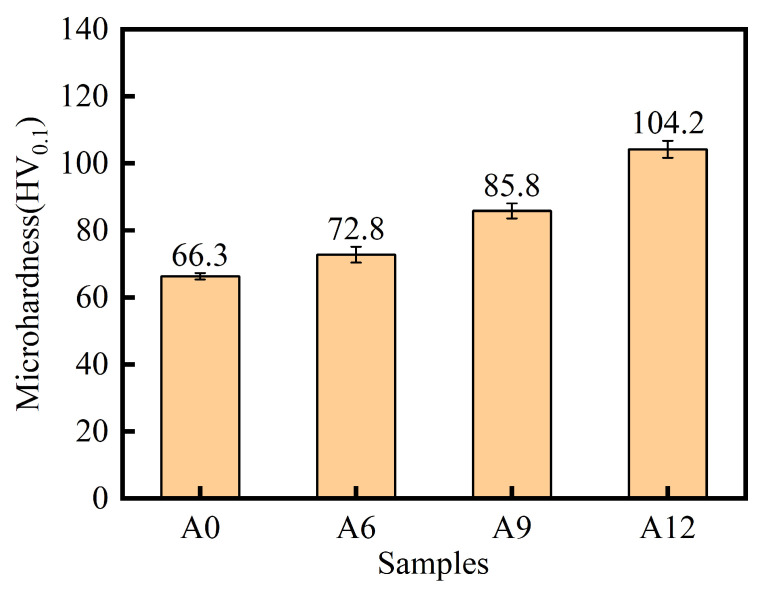
Microhardness of the samples with different compositions sintered at 1200 °C and subsequently processed by hot extrusion.

Since the specimens sintered and hot-extruded at 1200 °C exhibited the optimal tensile properties, microhardness and friction/wear tests were conducted at this temperature. Figure 7 shows the microhardness of samples with various compositions after being sintered at 1200 °C and undergoing hot extrusion treatment. It can be observed that the microhardness of the composite gradually increases with the rising content of ceramic particles. Specifically, the A12 sample achieves a hardness value of 104.2 HV_0.1_, demonstrating a significant improvement compared to the A0 group without ceramic particles. This enhancement is attributed to the high intrinsic hardness of the Al_2_O_3_ and TiC ceramic particles. Their distribution within the matrix contributes to an increased overall resistance to plastic deformation. Furthermore, a well-bonded interface formed between the particles and the matrix facilitates effective load transfer under external stress, allowing the ceramic particles to fully exert their load-bearing role. The combined effects collectively lead to the improved hardness of the composite [37,38].

**Figure 8:**
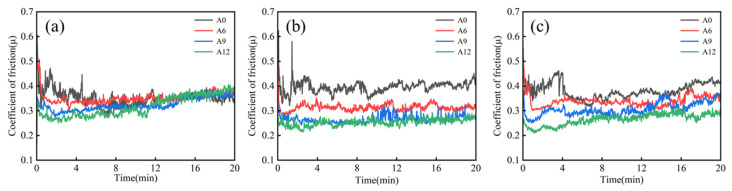
Relationship between the friction coefficient and sliding time under various loads for samples with different compositions sintered at 1200 °C and subsequently processed by hot extrusion: (**a**) 20 N, (**b**) 30 N, and (**c**) 40 N.

Figure 8 shows the relationship between the friction coefficient and sliding time under different applied loads for the samples with varying compositions sintered at 1200 °C followed by hot extrusion. The applied load exerts a notable influence on the stability of the friction behavior. As shown in Figure 8a, under a load of 20 N, the friction coefficients of all samples exhibit considerable fluctuations, indicating relatively unstable friction processes at this load. When the load is increased to 30 N (Figure 8b), samples A0, A6, and A9 still demonstrate a certain degree of instability, whereas sample A12 displays excellent frictional stability with a smooth curve and significantly reduced fluctuations. A further increase in the load to 40 N (Figure 8c) leads to noticeable fluctuations in the friction coefficients of all samples again, accompanied by a decline in stability. A comprehensive comparison reveals that the A12 sample exhibits optimal frictional stability under the 30 N load, characterized by a smooth friction coefficient curve without significant fluctuations [39].

**Figure 9:**
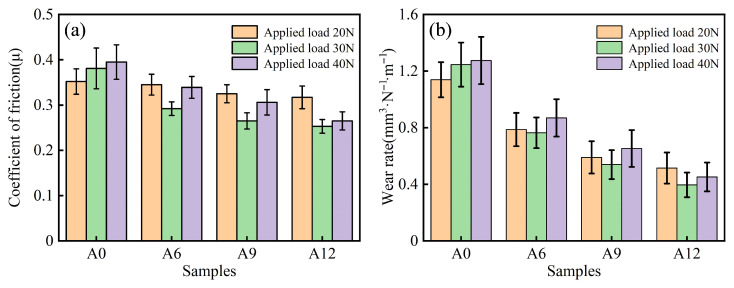
Samples of different compositions after sintering at 1200 °C and hot extrusion treatment under different loads: (**a**) Coefficient of friction; (**b**) Wear rate.

Figure 9 shows the coefficient of friction and wear rate of hot-extruded samples with varying compositions after sintering at 1200 °C, tested under different applied loads. As shown in Figure 9a, the friction coefficient of the composites decreases with increasing ceramic particle content under the same load. Regarding the influence of load, the friction coefficient of specimen A0 increases monotonically with increasing load. In contrast, the friction coefficients of specimens A6, A9, and A12 first decrease and then increase as the load increases. The friction coefficient of specimen A12 reaches its minimum value at a load of 30 N. Analysis of the wear rate (Figure 9b) indicates that the wear rates of all specimens show a significant decreasing trend under different loads. Specifically, the wear rate of sample A12 is the lowest at the 30 N load. This result is consistent with the described variations in friction behavior. The findings agree with the report by Jeyasimman et al., which confirmed that the hybrid (Al_2_O_3_ + TiC) composites possess lower wear rates and friction coefficients compared to AA 6061 [40].

**Figure 10:**
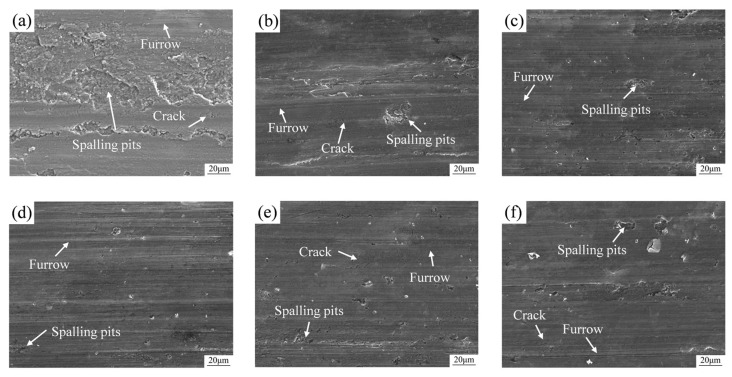
Wear surface morphology of samples with different compositions sintered at 1200 °C and subsequently processed by hot extrusion: (**a**) A0 30 N; (**b**) A6 30 N; (**c**) A9 30 N; (**d**) A12 30 N; (**e**) A12 20 N; (**f**) A12 40 N.

Figure 10 presents the wear surface morphology of samples with different compositions sintered at 1200 °C and subsequently subjected to hot extrusion treatment, under various applied loads. As shown in Figure 10a, severe wear occurred on the 6061 aluminum alloy, characterized by numerous spalling pits of varying sizes, along with ploughing grooves and microcracks. The wear mechanism is primarily delamination wear [41]. With the increase in ceramic particle content, the size and number of pits and microcracks on the wear surface gradually decrease (Figure 10b–d), indicating that the wear mechanism of the composites is jointly influenced by adhesive wear and abrasive wear. Further comparison of the wear morphology of A12 under different loads (Figure 10d–f) reveals that under a 20 N load, the wear surface of the A12 sample exhibits a small number of pits and microcracks, and the wear mechanism is controlled by mild adhesive wear and abrasive wear. Under a 30 N load, the wear tracks on the sample surface are uniformly distributed with fewer pits, and no obvious microcracks are observed; the wear mechanism is dominated by abrasive wear. Under a 40 N load, the wear surface displays more pits and microcracks, accompanied by deep and wide ploughing grooves, and the wear mechanism shifts to severe adhesive wear and abrasive wear [42].

**Figure 11:**
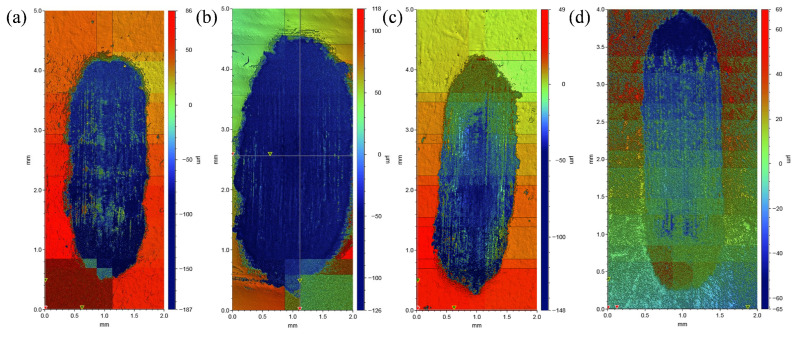
Three-dimensional surface morphology of the wear tracks for samples with different compositions sintered at 1200 °C and subsequently processed by hot extrusion, tested under a load of 30 N: (**a**) A0; (**b**) A6; (**c**) A9; (**d**) A12.

Figure 11 presents the three-dimensional morphology of the wear tracks on the surface of samples with different compositions, which were sintered at 1200 °C and subsequently processed by hot extrusion, under a 30 N applied load. As can be observed from the images, the wear track length for all four materials is approximately 4 mm. Regarding width, except for material A6 which has a wear track width of about 2 mm, the wear track widths of materials A0, A9, and A12 are all approximately 1 mm. By combining the images with the color scale on the right, further analysis of the surface morphological characteristics of the wear tracks can be conducted. The wear track areas of samples A0, A6, and A9 are predominantly blue. In contrast, sample A12 exhibits the least blue coverage in its wear track area, with a relatively uniform surface height distribution dominated by green and yellow regions. This reflects a relatively flat wear surface and the mildest degree of wear.

Furthermore, from the edge profiles of the wear tracks, samples A0, A6, and A9 show distinct annular depressions at the wear track edges, further indicating greater wear depth. In contrast, the wear track boundary of material A12 is smooth without noticeable depressions, which is consistent with its relatively uniform surface height distribution. The above results indicate that under a 30 N load, material A12 demonstrates superior wear resistance.

**Figure 12:**
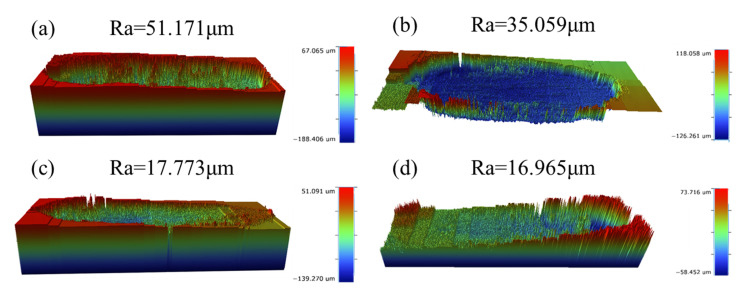
Three-dimensional surface topography of the wear scars on samples with different compositions sintered at 1200 °C and subsequently processed by hot extrusion, under a load of 30 N: (**a**) A0; (**b**) A6; (**c**) A9; (**d**) A12.

Figure 12 shows the three-dimensional contour topography and surface roughness (Ra) results of materials with different compositions under the action of 30 N. As can be seen from Figure 12a–d, the surface roughness of the samples gradually decreases with the increase in ceramic particle content. This trend is consistent with the wear rate tests and aligns with previous findings that surface roughness is positively correlated with the friction coefficient [43]. With reference to the color scale on the right, a large area of deep red is distributed on the surface of the A0 sample, indicating that the matrix endured significant stress during friction, resulting in severe and unstable wear. For A6 and A9 samples, their worn surfaces exhibit considerable red and blue areas, suggesting fluctuations in the wear morphology. In contrast, the grooves on the worn surface of the A12 sample show the smallest variation in depth. The surface is predominantly composed of green and yellow areas with lower height values, and almost no high-value red or blue areas are observed, further demonstrating its superior wear resistance.

## 4. Conclusions

In this study, Al_2_O_3_-TiC/6061Al composites were successfully fabricated via an in situ powder metallurgy route by incorporating TiO_2_ and graphite powders into a 6061 Al matrix. The microstructures, mechanical properties, and friction-wear behaviors of the composites were investigated. The main conclusions are summarized as follows:

(1) The microstructure of the composite varied with the sintering temperature. At 1000 °C and 1100 °C, structure consisted primarily of Al_3_Ti, Al_2_O_3_, and TiC particles, with slight agglomeration observed in localized areas. At 1200 °C and 1250 °C, the microstructure was predominantly composed of Al_2_O_3_ and TiC particles, which were uniformly distributed throughout the matrix without significant agglomeration.
(2) At a given sintering temperature, the tensile strength of the composites increased monotonically with the content of ceramic particles. As the sintering temperature rose, the tensile strength initially increased and then decreased. The A12 composite sintered at 1200 °C exhibited the optimal tensile properties, with a tensile strength of 246 MPa and an elongation of 12.7%, representing a 30.8% improvement in strength compared to the A0 sample while maintaining a relatively high ductility.
(3) Compared to the matrix, the hardness and wear resistance of the composites are significantly improved. The A12 sample exhibits the highest hardness (104.2 HV_0.1_), representing an increase of 36.3% compared to A0 (66.3 HV_0.1_). In terms of wear performance, under a 30 N load, A12 shows the lowest friction coefficient (0.263) and the lowest wear rate (0.396 mm^3^·N^−1^·m^−1^). Wear surface morphology analysis indicates that the wear mechanism of A0 is delamination wear, while that of the A12 composite is primarily abrasive wear.

## Figures and Tables

**Table 1:** Chemical composition of the 6061 Al alloy (mass fraction %).

Mg	Si	Fe	Cr	Ti	Mn	Zn	Al
1.05	0.52	0.24	0.03	0.02	0.08	0.03	Bal.

**Table 2:** The specific compositions of different samples.

Sample IDs	Sample Composition	6061 Al, wt%	TiO_2_, wt%	C, wt%
A0	6061 Al	100	0	0
A6	6061 Al-6% (TiO_2_ + C)	93.1	6	0.9
A9	6061 Al-9% (TiO_2_ + C)	90.8	8	1.2
A12	6061 Al-12% (TiO_2_ + C)	88.5	10	1.5

**Table 3:** Volume fraction of the reinforcement phase in samples with different compositions sintered at 1200 °C and 1250 °C followed by hot extrusion.

Sample IDs	Al_2_O_3_ vol%	TiC vol%
A0	0	0
A6	3.62	2.56
A9	4.89	3.46
A12	6.19	4.38

**Table 4:** The actual density, theoretical density, density and porosity of samples sintered at different temperatures and subjected to hot extrusion.

Samples	Actual Density (g/cm^3^)	Theoretical Density (g/cm^3^)	Density (%)	Porosity (%)
A0	2.65 ± 0.03	2.70	98.16	1.84
1200-A6	2.69 ± 0.03	2.80	96.13	3.87
1200-A9	2.71 ± 0.04	2.84	95.30	4.70
1200-A12	2.75 ± 0.05	2.91	94.51	5.49
1250-A6	2.68 ± 0.04	2.80	95.64	4.36
1250-A9	2.69 ± 0.04	2.84	94.64	5.36
1250-A12	2.68 ± 0.06	2.91	93.32	6.68

## Data Availability

The original contributions presented in this study are included in the article. Further inquiries can be directed to the corresponding authors.
